# Associations of Elevated Lipoprotein(a) with C-Reactive Protein and Type 2 Diabetes in a Multiethnic Population

**DOI:** 10.3390/biomedicines14091997

**Published:** 2026-09-05

**Authors:** Mohammad Qaddoumi, Ahmed N. Albatineh, Irina Al-Khairi, Preethi Cherian, Hamad Ali, Fahad Al-Ajmi, Fouz Alhaifi, Basil M. Alomair, Muhammad Abdul-Ghani, Fahd Al-Mulla, Jehad Abubaker, Mohamed Abu-Farha

**Affiliations:** 1 Pharmacology and Therapeutics Department, College of Pharmacy, Kuwait University, Kuwait City 13110, Kuwait; 2 Department of Translational Research, Dasman Diabetes Institute, Kuwait City 15462, Kuwait; fahad.alajmi@dasmaninstitute.org (F.A.-A.); fouz.alhaifi@dasmaninstitute.org (F.A.); abdulghani@uthscsa.edu (M.A.-G.); fahd.almulla@dasmaninstitute.org (F.A.-M.); mohamed.abufarha@dasmaninstitute.org (M.A.-F.); 3 Department of Biostatistics and Health Data Science, College of Health, Lehigh University, Bethlehem, PA 18015, USA; aha424@lehigh.edu; 4 Department of Biochemistry and Molecular Biology, Dasman Diabetes Institute, Kuwait City 15462, Kuwait; irina.alkhairi@dasmaninstitute.org (I.A.-K.); preethi.cherian@dasmaninstitute.org (P.C.); jehad.abubakr@dasmaninstitute.org (J.A.); 5 Department of Medical Laboratory Sciences, Faculty of Allied Health Sciences, Health Sciences Center (HSC), Kuwait University, Jabriya, PO Box 24923, Kuwait City 13110, Kuwait; hamad.ali@dasmaninstitute.org; 6 Division of Medicine, College of Medicine, Jouf University, Sakaka 72388, Saudi Arabia; bmalomair@ju.edu.sa; 7 Division of Diabetes, University of Texas Health Science Center, San Antonio, TX 78229, USA

**Keywords:** Type 2 diabetes, cardiovascular disease, lipoprotein (a), dyslipidemia, ethnic variation

## Abstract

**Background:** Lipoprotein(a) [Lp(a)] is a genetically determined, pro-atherogenic lipoprotein enriched in oxidized phospholipids and increasingly recognized as a key potential contributor to residual cardiovascular and inflammatory risk. However, data on its distribution and cardiometabolic associations in Middle Eastern populations remain limited. This study aimed to evaluate the prevalence of elevated Lp(a) and its association with type 2 diabetes (T2D), systemic inflammation, and ethnic variation in a multiethnic cohort. **Methods:** In this cross-sectional study, data from 2083 adults aged ≥ 18 years residing in Kuwait were collected through the Kuwait Diabetes Epidemiology Program (KDEP) at Dasman Diabetes Institute between 2011 and 2014 using random sampling. Plasma Lp(a) concentrations, cardiometabolic parameters, and high-sensitivity C-reactive protein (CRP) were measured. Elevated Lp(a) was defined as ≥ 50 mg/dL according to established clinical guidelines. Associations of elevated Lp(a) with T2D and CRP were assessed using multivariable Poisson regression models to estimate adjusted prevalence ratios. **Results:** The overall prevalence of elevated Lp(a) was 41.2%, exceeding global estimates. Significant ethnic variation was observed, with Arabs exhibiting the highest prevalence. After adjustment for potential confounders, individuals with T2D had a higher prevalence of elevated Lp(a) (APR = 1.166) and diabetes status significantly modified the association between CRP and elevated Lp(a) interaction (APR = 1.015), thus supporting a link between Lp(a) and systemic inflammation. **Conclusions:** Elevated Lp(a) was highly prevalent in this multiethnic Middle Eastern population and was independently associated with T2D and systemic inflammation. These findings support the emerging concept that Lp(a) may function as a molecular-inflammatory mediator potentially contributing to residual cardiovascular risk and highlight the importance of incorporating Lp(a) assessment into cardiometabolic risk stratification, particularly in high-risk populations.

## 1. Introduction

Diabetes mellitus is a chronic metabolic disease that poses major health problems and challenges worldwide. Currently, more than half a billion people are affected by diabetes and this number is projected to reach 1.3 billion by 2050 [1], imposing a substantial burden on society [2]. Type 2 diabetes (T2D), the most common form of diabetes, is characterized by persistent hyperglycemia due to inadequate insulin production by the pancreas, the lack of glucose utilization by tissues due to insulin resistance, or both. It is well known that T2D is a polygenic disease that can be inherited and is strongly associated with obesity and a sedentary lifestyle [3]. Furthermore, individuals with T2D are prone to developing microvascular complications such as nephropathy, neuropathy, and retinopathy, as well as macrovascular complications such as coronary artery disease and stroke [2].

Lipoprotein(a), often abbreviated as Lp(a), is a liver-assembled lipoprotein that is composed of an LDL-cholesterol particle with one apolipoprotein(a) (apo(a)) molecule that is covalently bound to apo-lipoprotein B-100 (apoB-100) via disulfide bonds [4]. Human apo(a) is a highly polymorphic glycoprotein that resembles the genetic sequence of plasminogen and contains a loop-like, typical structure called kringles. The kringle domain KIV2 is highly variable in size and may contribute up to 95% size heterozygosity within the population. A low number of the kringle domain KIV2 repeat sequence, rather than a high cholesterol content, is known to be associated with elevated Lp(a) plasma levels. Lp(a) plasma levels in humans are highly genetically inherited and may show remarkable variations between individuals up to threefold. Ethnic differences have been observed in circulating levels of Lp(a), where African Americans show substantially higher plasma levels of Lp(a) compared to Europeans and Asians [5]. Lifestyle changes, gender, and first-line lipid-lowering drugs have minimal effects on reducing Lp(a) levels. However, a low saturated fat diet, hormonal status, and renal or hepatic disease may exert modest effects [6].

Interest in Lp(a) grew recently after findings from Mendelian randomization and large observational studies indicated a robust independent association between elevated Lp(a) levels and risk of atherosclerotic cardiovascular disease (ASCVD) [7,8]. Lp(a) is considered more atherogenic and prothrombotic than other lipoproteins due to its propensity to carry oxidized phospholipids and its prolonged half-life [9]. Elevated Lp(a) levels are common worldwide, affecting approximately 20% of the population. Consequently, recent guidelines by the European Atherosclerosis Society, the American Heart Association (AHA), the American College of Cardiology (ACC), and the Multi-Society Cholesterol Guidelines have all considered Lp(a) levels ≥ 125 nmol/L (≥50 mg/dL) to be an independent risk factor for ASCVD, particularly among individuals with diabetes mellitus [10,11]. Furthermore, the risk of myocardial infarction and aortic valve stenosis increased with elevated Lp(a) levels [12,13]. Evidence indicates that individuals with ASCVD continue to face substantial residual cardiovascular risk despite treatment with maximally tolerated statin therapy. This persistent risk is largely attributed to residual cholesterol and inflammation that remain unaddressed by statins alone [14].

Although epidemiological studies have suggested an inverse relationship between Lp(a) levels and the incidence of T2D, large Mendelian randomization studies have not consistently confirmed this association [15]. Therefore, the relationship between Lp(a) and T2D remains unclear. Interestingly, among patients with T2D and established ASCVD, elevated Lp(a) has been identified as an independent predictor of recurrent cardiovascular events and adverse outcomes, irrespective of glycated hemoglobin (HbA1c) levels [16]. Similarly, elevated Lp(a) in patients with T2D has been associated with an increased risk of microvascular complications. Given that individuals with T2D already face a higher cardiovascular risk than those without diabetes, it is important to determine whether they also exhibit higher Lp(a) levels.

Middle Eastern populations, including those in the Gulf region, carry a disproportionately high burden of T2D and cardiovascular disease, driven by a combination of rapid urbanization, high rates of obesity, and elevated consanguinity, which increases the prevalence of genetically determined dyslipidemias such as elevated Lp(a) [17]. Although previous studies have evaluated Lp(a) in relation to diabetes, inflammation, and cardiovascular outcomes, evidence from Arab and broader Middle Eastern populations remains limited. Moreover, relatively few population-based studies have compared Arab, South Asian, and Southeast Asian adults residing within a common geographic and healthcare environment or evaluated whether diabetes status modifies the association between circulating CRP and elevated Lp(a). To our knowledge, this is among the first studies to examine simultaneously the interrelationships among Lp(a), systemic inflammation (assessed using CRP), and T2D across distinct ethnic subgroups within a single multiethnic Middle Eastern cohort, thereby addressing a significant gap in the regional cardiometabolic literature. Accordingly, this study aimed to evaluate the associations of elevated Lp(a) (≥50 mg/dL) with CRP and T2D status, and to explore potential ethnic variation in Lp(a) levels within a heterogeneous cohort of adults residing in Kuwait.

## 2. Materials and Methods

### 2.1. Participants, Study Design, and Ethical Approval

This is a cross-sectional study with data from the Kuwait Diabetes Epidemiological Program (KDEP), which recruited a representative sample of adults (aged ≥ 18 years) in Kuwait from 2011 to 2014 using random sampling with proportional allocation across Kuwait’s six administrative regions. The present analysis included 2083 participants with available stored plasma for Lp(a) measurement and the demographic and clinical information required for the planned analyses. Participants without an available Lp(a) measurement were excluded. Participants with known active infection or autoimmune disease, and those self-reporting the use of lipid-lowering, glucose-lowering, anti-inflammatory, or hormonal therapy were not excluded; and are thus already acknowledged as a limitation of our study. Detailed medication exposure data were not available with sufficient completeness to permit reliable medication-specific exclusion or adjustment. The study had ethical approval from the Ethical Review Committee at Dasman Diabetes Institute (Protocol number RA2011-003) and was conducted in accordance with the ethical standards of the Declaration of Helsinki. All participants provided a written informed consent before their study participation. The recruitment of the participants and their selection were discussed previously [18]. Participants were diagnosed with diabetes if they met the WHO criteria: FPG ≥ 7 mmol/L or HbA1c ≥ 6.5% (48 mmol/mol), as described previously [18].

### 2.2. Anthropometry, Physiological, and Laboratory Measurements

Anthropometric measurements for each participant included body weight, height, WC, HC, and BMI. BMI was calculated using the standard formula as follows: body weight (in kilograms) divided by the square of height (in meters). Vital signs such as blood pressure (BP) were measured using an Omron HEM-907XL Digital Sphygmomanometer. Blood samples were used to measure lipid and glycemic profiles, including FPG, HbA1c, fasting insulin, triglycerides (TG), total cholesterol (TC), low-density lipoprotein (LDL), and high-density lipoprotein (HDL). Vitamin D levels were quantified via measurement of the serum levels of its main metabolite, 25-hydroxyvitamin D (25(OH)D). Serum 25(OH)D levels were measured via chemiluminescent competitive immunoassay (CLIA) using a DiaSorin LIAISON analyzer (DiaSorin Inc, Stillwater, MN, USA) as described previously [19]. HsCRP secretion level was measured using ELISA kit (Biovendor, Asheville, NC, USA) and measured according to the manufacturer’s procedure as described previously [20].

Glucose and lipid profiles were assessed using the Siemens Dimension RXL chemistry analyzer (Diamond Diagnostics, Holliston, MA, USA), whereas HbA1c levels were determined using the VariantTM device (BioRad, Hercules, CA, USA). Insulin levels were quantified using the Access Ultrasensitive Insulin Assay (Beckman Coulter, Brea, CA, USA), with both intra- and inter-assay coefficients of variation not exceeding 6%. Insulin resistance was calculated using the HOMA-IR formula: (FBG in mmol/L) × (fasting insulin in mU/L)/22.5. For a detailed description of anthropometric, vital signs, and blood measurements, see the previous work [18].

Human Lp(a) in plasma was measured using the ELISA. Plasma samples stored at −80 °C were thawed on ice and centrifuged for 5 min at 10,000× *g* at 4 °C to remove any remaining cells or platelets and then used for the assay. Plasma level of Lp(a) was measured using the Lp(a) ELISA kit (an in-vitro diagnostic kit, Mercodia, Cat No. 10-1106-01, Uppsala, Sweden). ELISA was performed according to the manufacturer’s instructions. The calibrators provided in the kit are highly purified, fully validated commercial Lp(a), which were used in duplicates, and the standard curve was generated to calculate the concentration of Lp(a) in the samples. The samples were diluted 202X using the sample buffer as required by the kit protocol and their absorbance measured at 450 nm. The lowest detection limit as indicated in the kit is 0.07 U/L before application of the sample-dilution factor and no measurable cross-reactivity with plasminogen or apolipoprotein B. The intra-assay coefficient of variation was 1.0–5.0% while the inter-assay coefficient of variation was <10%. The kit reports concentrations in U/L and is calibrated against a purified, validated commercial Lp(a) preparation. Results were converted to mg/dL using the assay-specific factor supplied by the vendor, 1 U/L = 0.1254 mg/dL. Thus, the threshold of 50 mg/dL corresponded to approximately 399 U/L. This is an assay-specific conversion and should not be interpreted as a universal conversion between Lp(a) mass and particle concentration.

### 2.3. Statistical Analysis

Data were analyzed using STATA version 14 (STATA Corp, College Station, TX, USA). Data were checked for abnormalities, coded, and labeled accordingly. Continuous variables were summarized as median (interquartile range) rather than mean (SD) due to their skewed distribution; the median and IQR provide robust, outlier-resistant estimates of central tendency and spread that were considered more appropriate for the between-group comparisons presented. To test for a significant difference between a continuous covariate and a binary variable, the two-sample *t*-test was used if normality in both groups was satisfied; otherwise, the Mann–Whitney U test was used. To test differences in an outcome across a categorical exposure, ANOVA was used if normality and homogeneity of variances were satisfied; otherwise, the nonparametric Kruskal–Wallis test was used. To measure the strength of the linear relationship between two continuous variables, the Pearson correlation coefficient was calculated if both variables were normally distributed; otherwise, the Spearman rank correlation was calculated.

Rather than using a multiple logistic regression model to investigate the relationship between a binary outcome and a set of covariates when the outcome is prevalent, Poisson regression with a log link and a robust variance-covariance matrix of the estimators (VCE) was used to estimate a prevalence ratio (PR) or risk ratio. This approach is necessary because the literature shows that logistic regression odds ratios (OR) overestimate effect size when the outcome (Lp(a) ≥ 50 mg/dL) is prevalent (>10%). Finally, the effect modification (interaction) by diabetes status on the association between CRP and LP(a) was tested and estimated. This will highlight the effect of diabetes on the CRP and LP(a) association. In case this interaction was significant, it is customary to interpret the interaction effect rather than the main effects of CRP and diabetes status. All tests were two-tailed, and significance was set at 5%.

## 3. Results

### 3.1. Circulating Lp(a) Concentrations

**Figure 1:**
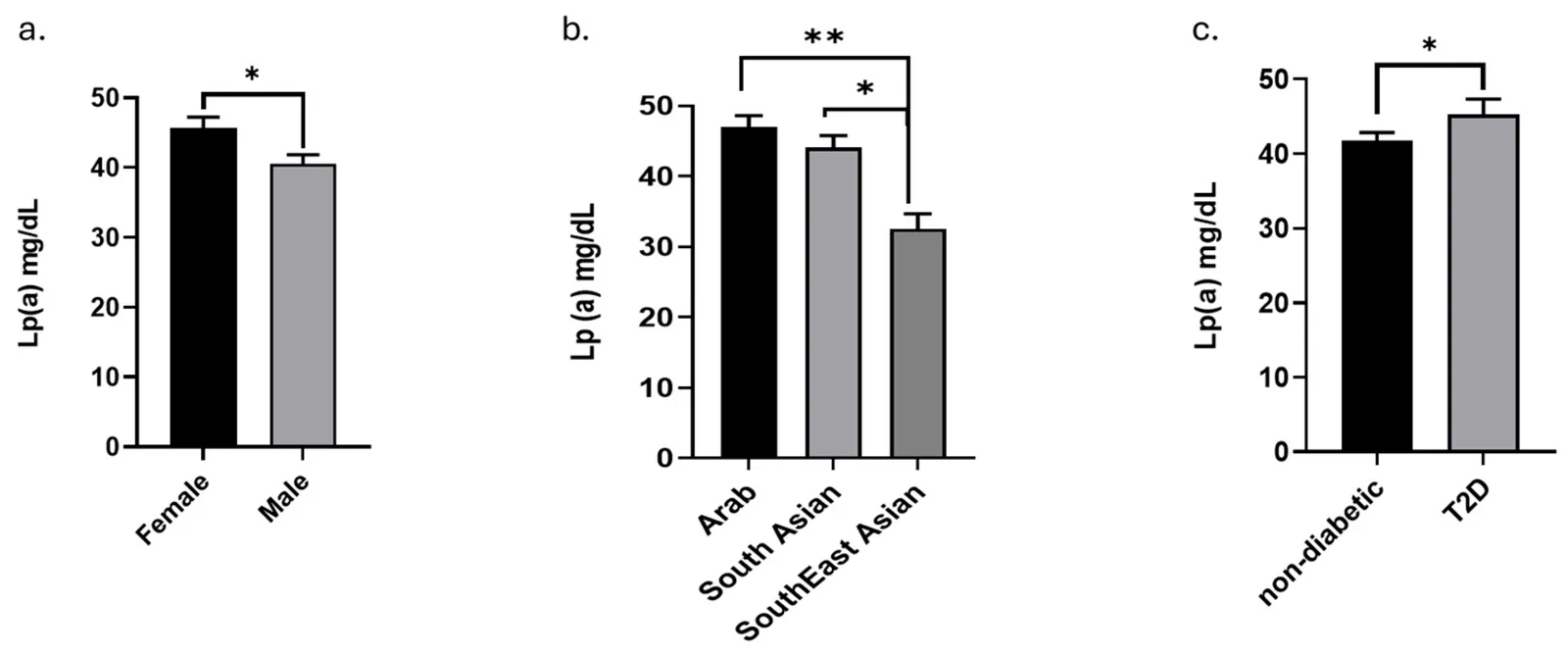
Circulation levels of Lp(a) in the entire study population stratified by (**a**). gender, (**b**). ethnicity, and (**c**). diabetes status. Data are presented as mean ± standard error. * *p* < 0.05, ** *p* < 0.01.

The mean Lp(a) level in the population under study was 42.76 ± 1 mg/dL. When the population was categorized based on gender, women showed higher levels of Lp(a) as compared to men (45.65 ± 1.59 mg/dL vs. 40.55 ± 1.27 mg/dL; *p*-value> 0.05; Figure 1a). When stratified based on ethnicity, Arabs showed the highest levels of Lp(a) (46.94 ± 1.63 mg/dL) followed by South Asians (44.05 ± 1.71 mg/dL) and Southeast Asians (32.53 ± 2.17 mg/dL; Figure 1b). To understand the effect of diabetes on the circulating levels of Lp(a) the cohort was divided into individuals with T2D and non-diabetic. The mean Lp(a) levels among individuals with T2D was higher (45.22 ± 2.11 mg/dL) when compared with those without diabetes (41.75 ± 1.12 mg/dLl Figure 1c).

### 3.2. Participants Characteristics According to Lp(a) Category

The final sample size included in the analysis was N = 2083. Descriptive analysis indicated that patients were mostly males (55.7%), non-Kuwaiti (70.2%), and had a median age of 45 years, with 36% less than 40 years old. Furthermore, 46.6% were of Arab ethnicity, 34.5% were South Asian, and 18.9% were Southeast Asian. Approximately 30.8% were T2D, 40.2% were overweight, and 38.6% were obese. Furthermore, according to the Homeostasis Model Assessment for Insulin Resistance (HOMA-IR) score, 49.7% were insulin resistant, with more details shown in Table 1.

If we adopt the dichotomization of Lp(a) into Normal (Lp(a) < 50 mg/dL) and High (Lp(a) ≥ 50 mg/dL), the results in Table 2 reveal that the prevalence of high Lp(a) was approximately 41.2% (n = 858). When categorical variables were cross-tabulated with Lp(a) levels, results indicated a significant association between gender and Lp(a) levels (*p*-value = 0.016), with 44.14% of females having higher Lp(a) levels compared to 38.85% among males. There was a significant association between ethnicity and Lp(a) levels (*p* < 0.001), with 47.6% of Arabs exhibiting elevated Lp(a) compared to 34.8% of South Asians and 44.0% of Southeast Asians. Diabetes status was also significantly associated with Lp(a) levels (*p* < 0.001), as 48.0% of individuals with T2D had elevated Lp(a) compared to 38.3% of non-diabetic participants. In contrast, age (categorized into tertiles), HOMA-IR score, and estimated glomerular filtration rate (eGFR) were not significantly associated with Lp(a) levels. CRP concentrations were compared between the Lp(a) < 50 mg/dL and Lp(a) ≥ 50 mg/dL groups after stratification by diabetes status. Among participants without diabetes, CRP did not differ significantly between the two Lp(a) categories (*p* = 0.796). Among participants with T2D, CRP was higher in the elevated-Lp(a) group than in the <50 mg/dL group (*p* < 0.001), as shown in Table 2.

For continuous covariates, the results indicated that the medians of HbA1c, aspartate aminotransferase (AST), C-reactive protein (CRP), Vitamin D, fasting plasma glucose (FPG), and systolic blood pressure (SBP) were significantly different across Lp(a) levels. In contrast, there was no significant difference in the medians of creatinine, insulin, hip circumference (HC), waist circumference (WC), and diastolic blood pressure (DBP) across levels of Lp(a) levels, with details in Table 2.

### 3.3. Univariable and Multivariable Associations with Elevated Lp(a) and Clinical Markers

To model and assess the association between high Lp(a) levels and several clinical covariates, Modified Poisson regression with log link function and robust variance-covariance estimates procedure was implemented. Through the univariate analysis presented in Table 3, males were found to have a 12% lower prevalence of elevated Lp(a) levels (PR = 0.879, *p* = 0.014), while South Asians had a 27% lower prevalence of elevated Lp(a) levels (PR = 0.731, *p* < 0.001). Both variables were independently and significantly associated with high Lp(a) levels. On the other hand, having T2D (PR = 1.255, *p*-value < 0.001), elevated levels of CRP (PR = 1.012, *p*-value < 0.001), and Vitamin D (PR = 1.009, *p*-value < 0.001) were independently and significantly associated with an increased prevalence of high Lp(a) levels.

**Figure 2:**
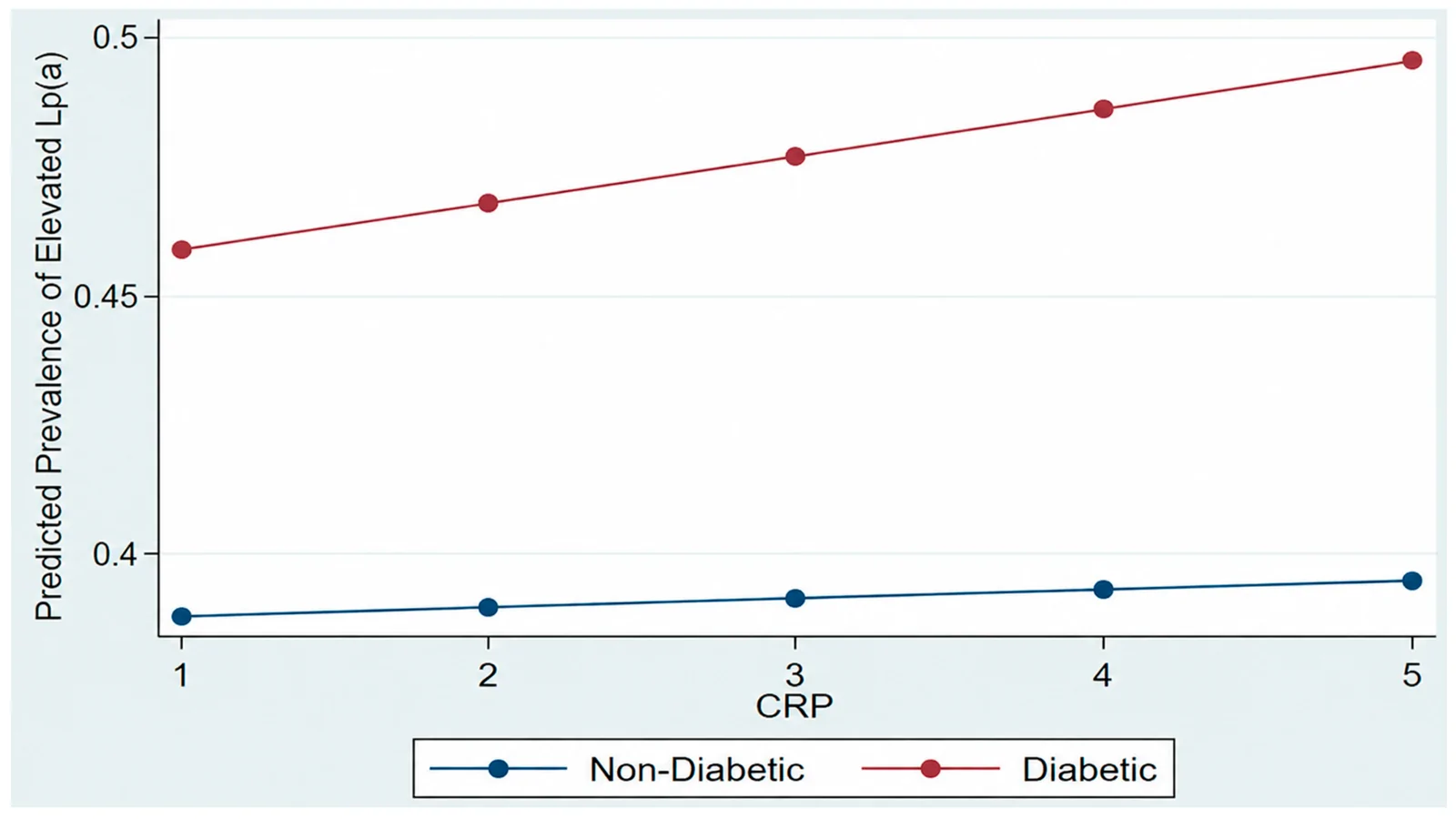
Marginal plot showing the effect modification of diabetes status on the association between CRP and prevalence of high Lp(a) after adjusting for covariates in Table 3.

To account for potential confounding factors, a multivariable-adjusted model was implemented. The results revealed that South Asians had a 27% lower prevalence of elevated Lp(a) compared to Arabs (APR = 0.720, *p* < 0.001), after adjusting for all other covariates. In comparison, a one-unit increase in Vitamin D was associated with a 0.8% increase in the prevalence of high Lp(a) level (APR = 1.008, *p*-value = 0.004) when all other covariates are held constant. On the other hand, age was inversely associated with the prevalence of high Lp(a) level (APR = 0.993, *p*-value = 0.014), showing a 1% decrease in the prevalence with each one-year increase in age. Also, a one-unit increase in total cholesterol was associated with a 5.5% increase in the prevalence of high Lp(a) level, independent of all other covariates. Finally, diabetes status was shown to be an effect modifier (significant interaction) for the association between CRP and Lp(a) level. This means that the association between CRP and Lp(a) was dependent on diabetes status. Results indicated that a one-unit increase in CRP would result in a 1.5% increase in the prevalence of high Lp(a) among diabetic patients compared to non-diabetic patients, after adjusting for other covariates presented in Table 3. The 95% confidence interval for the exponentiated coefficient was 1.000–1.030, and the interaction *p*-value was 0.043. This clearly underscores the effect of diabetes on the association between CRP and Lp(a). The magnitude of the interaction was modest and should not be interpreted as a 1.5-percentage-point absolute increase or as evidence of causation. The impact of diabetes status on this association was depicted graphically in Figure 2.

### 3.4. Lp(a) Distribution Among Participants with T2D

To clarify the role of diabetes in the relationship between Lp(a) levels and other covariates, subgroup analyses were conducted among individuals with T2D only. The results showed that ethnicity remained significantly associated with Lp(a) levels (Table 4). Among participants with T2D, elevated Lp(a) was present in 62.5% of Arabs compared to 31.1% and 19.7% among South Asian and Southeast Asian participants, respectively. Additionally, significant differences were observed between individuals with normal and elevated Lp(a) in body mass index (BMI), AST, CRP, FPG, WC, HC, SBP, and DBP. In contrast, gender, HOMA-IR, and eGFR were not significantly associated with Lp(a) levels. This stratified analysis also revealed that both WC and HC were significantly higher among patients with elevated Lp(a), while HbA1c levels were marginally higher (*p* = 0.057) in those with elevated Lp(a), as summarized in Table 4.

## 4. Discussion

This study provides new insight into the relationship between lipoprotein(a), T2D, inflammation, and ethnicity in a large multiethnic cohort from Kuwait. The principal findings were a high prevalence of elevated Lp(a) in this population, marked ethnic variation with the greatest burden among Arabs, and an independent association between elevated Lp(a) and T2D after multivariable adjustment. Furthermore, diabetes status was found to be a significant effect modifier for the association between CRP and Lp(a). These findings support the concept that Lp(a) is not merely a passive lipid biomarker, but rather a genetically determined, proatherogenic, and proinflammatory lipoprotein particle that may contribute to residual cardiovascular risk in high-risk metabolic populations [7,21,22].

### 4.1. Lp(a) as a Molecular Driver of Inflammation and Atherogenesis

Lp(a) possesses unique structural and functional properties that distinguish it from other apoB-containing lipoproteins. It consists of an LDL-like particle covalently bound to apolipoprotein(a), whose kringle IV type 2 repeat polymorphism largely determines circulating concentrations [4].

A major mechanistic implication of the present work is the observed modest but statistically significant association between elevated Lp(a) and CRP, which supports the concept of an Lp(a)-linked inflammatory axis. Experimental work has shown that Lp(a) can induce inflammatory responses in human monocytes, including increased interleukin-6 signaling, whereas oxidized phospholipids carried by Lp(a) can promote arterial wall inflammation and inflammatory monocyte responses [23,24]. Since IL-6 is a key upstream driver of hepatic CRP synthesis via the JAK/STAT3 pathway activation, the modest association observed here between Lp(a) and CRP is biologically plausible and consistent with a model in which Lp(a) amplifies vascular and systemic inflammatory signaling. Recent lipidomic and functional studies have also identified diacylglycerols and lysophosphatidic acid enriched on Lp(a) as potential mediators of monocyte activation and inflammatory cytokine secretion, including IL-6, suggesting lipid species beyond oxidized phospholipids may contribute to Lp(a)-associated vascular inflammation [25]. This interpretation is consistent with the broader concept of residual inflammatory risk, whereby individuals remain at substantial cardiovascular risk despite LDL-C lowering because inflammation persists as an independent pathogenic pathway [7,14]. T2D is accompanied by hyperglycemia, oxidative stress, endothelial dysfunction, and chronic low-grade inflammation, which could plausibly intensify inflammatory responses to oxidized phospholipid-rich particles. However, oxidized phospholipids, IL-6, endothelial activation, and oxidative stress were not measured in this study. The unexpected positive association between vitamin D and elevated Lp(a) observed in our cohort remains biologically uncertain and may reflect residual confounding by diet, sun exposure, renal or hepatic function, or supplementation status, none of which were captured in this study. This finding should therefore be considered exploratory and requires independent replication.

### 4.2. Lp(a), Type 2 Diabetes, and Cardiometabolic Risk Amplification

The association between elevated Lp(a) and T2D in our adjusted analysis is clinically important. Individuals with T2D already carry markedly increased cardiovascular risk, and previous cohort studies have shown that elevated Lp(a) is associated with worse long-term cardiovascular outcomes in this group [16,26]. In the ARIC analysis, Lp(a) was associated with cardiovascular events among individuals with diabetes or prediabetes, supporting its role as a risk-enhancing factor in dysglycemic populations [11]. Likewise, prospective data in patients with T2D have shown that elevated Lp(a) predicts recurrent adverse cardiovascular outcomes, and pooled multiethnic cohort analyses suggest that high Lp(a) may confer particularly pronounced ASCVD risk in individuals with T2D [16,26]. These findings therefore reinforce the potential clinical value of measuring Lp(a) in individuals with diabetes when refining residual cardiovascular risk beyond standard glycemic and lipid markers.

The biological basis of the relationship between Lp(a) and T2D remains complex. Some epidemiological and genetic studies suggest an inverse or inconsistent association between Lp(a) concentration and incident diabetes, whereas other observational datasets indicate that elevated Lp(a) remains clinically harmful once T2D is established [16,26,27]. In a pooled analysis of five prospective US cohorts, elevated Lp(a) predicted long-term ASCVD across racial and ethnic groups, with a stronger association observed among participants with diabetes [27]. One possible interpretation is that the diabetogenic relationship and the vascular risk relationship are not identical: Lp(a) may not uniformly increase diabetes incidence across all settings, yet among patients who already have T2D, elevated Lp(a) may still serve as a major enhancer of vascular inflammation, thrombogenicity, and atherosclerotic burden. High-sensitivity CRP is the most widely validated, clinically accessible, and guideline-endorsed marker of systemic/vascular inflammation and residual inflammatory risk utilized in major cardiovascular outcome trials [28]. Interleukin-6 (IL-6) and other inflammatory markers were not available in this cohort and are mentioned as limitations of our study. A recent analysis of UK Biobank data and secondary-prevention populations from two clinical trials found that higher Lp(a) was associated with cardiovascular events regardless of baseline CRP, suggesting that Lp(a) and systemic inflammation represent largely independent but clinically complementary risk pathways [29]. Our finding that diabetes status modified the positive association between CRP and elevated Lp(a) together with evidence of worse cardiovascular outcomes in individuals with diabetes and prediabetes, supports the clinical relevance of elevated Lp(a) for cardiovascular risk stratification in dysglycemic populations [30].

### 4.3. Ethnic Variation and Genetic Determinants

Another notable finding was the substantial ethnic heterogeneity in Lp(a) distribution, with Arabs showing the highest prevalence of elevated Lp(a) of 47.6% in this cohort (median Lp(a) of 32.4 mg/dL). Ethnic variation in Lp(a) is well established and is largely explained by differences in LPA genetics, apo(a) isoform size, and allele distribution across populations [5,22,31]. Although previous studies have reported substantial differences among ethnic groups, data from Arab populations remain limited [5,31]. In this context, our study therefore adds evidence from the Gulf region and suggests that inherited Lp(a)-mediated cardiovascular risk may be underrecognized in Middle Eastern populations. Ethnic differences in inflammatory phenotype may further compound this risk; for instance, a higher prevalence of the haptoglobin 2-2 phenotype, which has been linked to elevated CRP levels, has been reported and may contribute to an ethnicity-specific synergism between inflammation, T2D, and Lp(a) in cardiovascular disease [32]. However, neither haptoglobin genotype nor circulating haptoglobin was measured in our cohort. The lack of association between Lp(a) levels and waist-to-hip ratio, BMI, or insulin resistance observed in this cohort further supports that elevated Lp(a) levels, especially in Arabs, may be primarily genetically determined rather than influenced by diet or lifestyle factors. Given the high rates of cardiometabolic disease and consanguinity in the region, these observations support the need for further studies incorporating LPA genotyping, apo(a) isoform characterization, and longitudinal cardiovascular follow-up.

### 4.4. Clinical Implications and Emerging Therapeutics

The clinical implications of this study are timely. Contemporary statements and guidelines now recognize elevated Lp(a) as an important inherited cardiovascular risk factor and support at least once-in-a-lifetime measurement in appropriate individuals, particularly those with premature ASCVD, family history, or unexplained residual risk [4,24]. Our data suggest that this recommendation may be especially relevant for Arabs and individuals with T2D, in whom the prevalence and potential impact of elevated Lp(a) may be greater. Individuals with T2D are already at increased absolute cardiovascular risk, but this risk varies substantially and is not captured completely by HbA1c, LDL cholesterol, blood pressure, or conventional risk scores. Measurement of Lp(a) can identify a largely inherited component of risk that is not included in a routine lipid profile and is minimally modified by lifestyle. This is particularly important because conventional lifestyle measures and most standard lipid-lowering therapies have a limited effect on Lp(a) concentrations, leaving a substantial component of risk untreated [22,33]. In patients with T2D, knowledge of Lp(a) may be particularly useful when cardiovascular risk appears disproportionate to conventional risk factors or when there is premature or recurrent ASCVD.

This issue is increasingly relevant because the therapeutic landscape for Lp(a) is rapidly evolving. While PCSK9 inhibitors produce modest reductions in Lp(a), newer targeted approaches, including antisense oligonucleotides, small interfering RNA therapies, and small-molecule inhibitors, have shown markedly greater Lp(a)-lowering efficacy in clinical development [33]. Agents such as pelacarsen, olpasiran, lepodisiran, and muvalaplin have generated major interest because they may finally provide a mechanism-based strategy for addressing Lp(a)-mediated potential residual risk, particularly in individuals already receiving statin therapy. Although definitive cardiovascular outcome data are still pending, our findings highlight the potential value of identifying populations that may derive particular benefit from future Lp(a)-targeted interventions.

This study advances current knowledge in three principal ways. First, it provides population-based Lp(a) data from an underrepresented Gulf population with a substantial cardiometabolic disease burden. Second, it permits direct comparison of Lp(a) distributions among Arab, South Asian, and Southeast Asian participants recruited within a common setting. Third, it evaluates diabetes as a potential effect modifier of the association between CRP and elevated Lp(a), rather than considering diabetes and inflammation only as independent covariates. These analyses may help generate hypotheses for longitudinal and molecular studies of Lp(a)-associated cardiovascular risk in ethnically diverse populations.

### 4.5. Strengths and Limitations

The present study has several strengths, including its large sample size, inclusion of multiple ethnic groups, and the use of Poisson regression with robust estimates of the variance-covariance matrix to estimate prevalence ratios (more intuitive) for a common outcome than odds ratios. These features strengthen the internal validity and translational relevance of the findings. However, several limitations should be acknowledged. First, the cross-sectional design precludes causal inference. Second, although we provide biologically plausible mechanistic interpretations, we did not directly measure LPA variants, apo(a) isoform size, oxidized phospholipids, IL-6, or other inflammatory mediators, which limits mechanistic resolution. Third, some potentially residual confounders in participants such as the presence of known active infection or immune disease, exposure to medications (statins, PCSK9-directed therapy, oral contraceptives, hormone replacement therapy, antidiabetic agents, and anti-inflammatory drugs), menopausal status, and detailed dietary factors were not available in our study and may have influenced Lp(a), CRP, and their observed associations in patients with T2D. Fourth, cardiovascular events were not assessed; consequently, the study did not directly measure residual cardiovascular risk or demonstrate that the observed associations translate into clinical outcomes. Finally, the findings may not be generalizable to all Middle Eastern populations, particularly populations with different ancestry or healthcare characteristics. Accordingly, future work should include prospective cohort follow-up, multi-omics characterization, and molecular phenotyping to better define the pathways linking Lp(a), inflammation, and cardiometabolic disease.

### 4.6. Conclusions

In conclusion, elevated Lp(a) was highly prevalent in this multiethnic population, particularly among Arabs, and was independently associated with T2D and systemic inflammation. Diabetes status modified the association between CRP and elevated Lp(a). These findings support the emerging paradigm of Lp(a) as a molecular-inflammatory lipid mediator potentially contributing to residual cardiovascular risk beyond traditional lipids. Routine assessment of Lp(a), especially in high-risk ethnic and diabetic populations, may improve cardiometabolic risk stratification and help identify individuals who could benefit from future targeted therapies and improve their cardiometabolic outcomes.

## Figures and Tables

**Table 1:** Characteristics of the study participants across some covariates (N = 2083).

Covariate	Statistic ^†^	Covariate	Statistic ^†^
Gender, n (%)		Diabetes Status, n (%)	
Male	1161 (55.7)	Non-Diabetic	1425 (69.2)
Female	922 (44.3)	T2D	633 (30.8)
Age, (Tertiles), n (%)		BMI, n (%)	
LT 40	750 (36.0)	Underweight/Normal (LT 24.99)	441 (21.2)
40–50	680 (32.7)	Overweight (25–29.9)	837 (40.2)
>50	653 (31.3)	Obese (≥30)	805 (38.6)
Ethnicity, n (%)		HOMA score, n (%)	
Arab	899 (46.6)	Normal (≤2)	969 (50.3)
South Asian	666 (34.5)	IR (>2)	958 (49.7)
Southeast Asian	364 (18.9)		
HC, median (IQR)	102.3 (13)	WC, median (IQR)	95 (15)
SBP mmHg, median (IQR)	131 (26)	DBP mmHg, median (IQR)	80 (16)
Age, median (min, max)	45 (18, 82)	FPG mmol/L, median (IQR)	5.3 (1.7)
HbA1c %, median (IQR)	5.8 (1.2)	Insulin, median (IQR)	7.9 (6.7)
TC mmol/L, median IQR)	5.1 (1.33)	Vitamin D nmol/L, median (IQR)	12.01 (10.8)
AST U/L, median (IQR)	21 (8)	ALT U/L, median (IQR)	37 (19)
CRP µg/dL, median (IQR)	3 (2)	Creatinine µmol/L, median (IQR)	76 (24)
Lp(a) mg/dL, median (IQR)			
Arab	32.4 (48.7)		
South Asian	28.9 (40.4)		
Southeast Asian	21.2 (30.9)		

^†^ The total may not add up to N due to missing values, PR: Prevalence Ratio, APR: Adjusted Prevalence Ratio.

**Table 2:** Clinical characteristics according to Lp(a) category (N = 2083).

Covariate	N ^†^	Lp(a) Category		*p*-Value
		Normal (<50 mg/dL)	High (≥50 mg/dL)	
Gender, n (%)				0.016 ^a^
Male	1161	710 (61.15)	451 (38.85)	
Female	922	515 (55.86)	407 (44.14)	
Age, (Tertiles), n (%)				0.514 ^a^
LT 40	750	447 (59.60)	303 (40.40)	
40–50	680	406 (59.71)	274 (40.29)	
>50	653	372 (56.97)	281 (43.03)	
Ethnicity, n (%)				<0.001 ^a^
Arab	899	471 (52.39)	428 (47.61)	
South Asian	666	434 (65.17)	232 (34.83)	
Southeast Asian	364	204 (56.04)	160 (43.96)	
Diabetes Status, n (%)				<0.001 ^a^
Non-Diabetic	1425	880 (61.75)	545 (38.25)	
T2D	633	329 (51.97)	304 (48.03)	
BMI, (median) (IQR)	2080	(28.2) (6.9)	(28.4) (7.5)	0.773 ^b^
HOMA score, n (%)				0.433 ^a^
Normal (≤2)	971	568 (58.50)	403 (41.50)	
IR (>2)	956	542 (56.69)	414 (43.31)	
Age, median (IQR)	2082	44 (16)	45 (16)	0.239 ^b^
HbA1c %, median (IQR)	2012	5.8 (1.02)	5.8 (1.6)	0.021 ^b^
TC mmol/L, median (IQR)	2013	5.1 (1.37)	5.19 (1.4)	0.094 ^b^
AST U/L, median (IQR)	1931	22 (9)	21 (7)	0.030 ^b^
CRP µg/dL, median (IQR)	1915	3 (2)	3 (3)	0.011 ^b^
CRP µg/dL, median (IQR)				
Non-diabetic	1266	3 (2)	3 (2)	0.796 **^b^**
Diabetic	626	3 (2)	3 (5)	<0.001 **^b^**
Creatinine µmol/L, median (IQR)	1932	76 (25)	76 (23)	0.790 ^b^
Vitamin D nmol/L, median (IQR)	1932	11.21 (9.82)	13.22 (11.62)	<0.001 ^b^
FPG mmol/L, median (IQR)	2011	5.2 (1.4)	5.4 (2.1)	0.001 ^b^
Insulin, median (IQR)	1927	7.97 (6.32)	7.8 (7.1)	0.600 ^b^
HC, median (IQR)	1926	102 (13)	103 (15)	0.926 ^b^
WC, median (IQR)	2005	95 (14.5)	95 (16)	0.349 ^b^
SBP mmHg, median (IQR)	2013	132 (27)	130 (25)	0.045 ^b^
DBP mmHg, median (IQR)	2013	80 (17)	79 (16)	0.388 ^b^

^a^ Based on Fisher’s exact test, ^b^ based on Wilcoxon rank sum test, ^†^ The total may not add up to N due to missing values.

**Table 3:** Associations of elevated Lp(a) with demographic and clinical variables estimated using modified Poisson regression with robust variance estimates (N = 2083).

Covariate	Univariate Analysis PR (95%CI) (*p*-Value)	Adjusted Analysis APR (95%CI) (*p*-Value)
Gender		
Female	Ref	Ref
Male	0.879 (0.794, 0.9748) (0.014)	0.966 (0.863, 1.081) (0.545)
Ethnicity		
Arab	Ref	Ref
South Asian	0.731 (0.646, 0.828) (<0.001)	0.720 (0.628, 0.825) (<0.001)
Southeast Asian	0.923 (0.806, 1.056) (0.246)	0.872 (0.745, 1.019) (0.085)
Diabetes Status		
Non-Diabetic	Ref	Ref
T2DM	1.255 (1.131, 1.394) (<0.001)	1.166 (1.011, 1.343) (0.034)
Age (years)	1.002 (0.997, 1.007) (0.387)	0.993 (0.988, 0.998) (0.013)
HC (cm)	1.002 (0.998, 1.006) (0.348)	0.995 (0.990, 1.000) (0.064)
TC (mmol/L)	1.036 (0.990, 1.084) (0.121)	1.055 (1.004, 1.108) (0.034)
CRP (µg/dL)	1.012 (1.005, 1.019) (<0.001)	1.004 (0.992, 1.016) (0.479)
Vitamin D (nmol/L)	1.009 (1.004, 1.014) (<0.001)	1.008 (1.003, 1.013) (0.003)
Diabetes Status x CRP ‡		
Non-Diabetic	Ref	Ref
T2DM	1.015 (1.001, 1.029) (0.039)	1.015 (1.000, 1.030) (0.043)

PR: Prevalence Ratio, APR: Adjusted Prevalence Ratio. ‡ Univariate analysis results are from the model that includes diabetes status, CRP, and the interaction term.

**Table 4:** Descriptive analysis of Lp(a) distribution among individuals with T2D only across clinical covariates (N = 633).

Covariate	N ^†^	Lp(a) Level		*p*-Value
		Normal (<50)	High (≥50)	
Gender, n (%)				0.371 ^a^
Male	381	204 (53.5)	177 (46.5)	
Female	252	125 (49.6)	127 (50.4)	
Age, (Tertiles), n (%)				0.112 ^a^
LT 40	76	48 (63.2)	28 (36.8)	
40–50	194	99 (51.0)	95 (49.0)	
>50	363	182 (50.1)	181 (49.9)	
Ethnicity, n (%)				<0.001 ^a^
Arab	365	137 (37.5)	228 (62.5)	
South Asian	206	142 (68.9)	64 (31.1)	
Southeast Asian	61	49 (80.3)	12 (19.7)	
BMI, n (%)				0.059 ^a^
Underweight/Normal (LT 24.99)	81	42 (51.9)	39 (48.1)	
Overweight (25–29.9)	218	127 (58.3)	91 (41.7)	
Obese (≥ 30)	334	160 (47.9)	174 (52.1)	
BMI, (median) (IQR)	631	29.7 (7.1)	31 (8.5)	0.017 ^b^
HOMA score, n (%)				0.543 ^a^
Normal (≤2)	119	65 (54.6)	54 (45.4)	
IR (>2)	511	263 (51.5)	248 (48.5)	
HbA1c %, median (IQR)	633	7.4 (2.3)	7.7 (2.7)	0.057 ^b^
TC mmol/L, median (IQR)	632	5.0 (1.5)	5.0 (1.55)	0.612 ^b^
AST U/L, median (IQR)	631	22.0 (10)	21 (9.0)	0.012 ^b^
CRP µg/dL, median (IQR)	626	3 (2)	3 (5)	<0.001 ^b^
Creatinine µmol/L, median (IQR)	632	78 (21)	77 (23)	0.471 ^b^
Vitamin D nmol/L, median (IQR)	632	12.82 (12.01)	12.49 (14.34)	0.842 ^b^
FPG mmol/L, median (IQR)	632	7.5 (3.2)	8.0 (4.20)	0.025 ^b^
Insulin, median (IQR)	630	10.18 (9.32)	10.55 (10.36)	0.468 ^b^
HC, median (IQR)	628	104 (14)	107 (18.75)	0.018 ^b^
WC, median (IQR)	630	99 (16.75)	101 (16.5)	0.027 ^b^
SBP mmHg, median (IQR)	632	140 (27)	135 (23)	<0.001 ^b^
DBP mmHg, median (IQR)	632	83 (15)	81 (14)	0.003 ^b^

^a^ Based on Fisher’s exact test, ^b^ based on Wilcoxon rank sum test, ^†^ The total may not add up to N due to missing values.

## Data Availability

The datasets used and/or analyzed during this study are available from the corresponding author on reasonable request.

## Abbreviations

The following abbreviations are used in this manuscript:

ACC	American college of cardiology
AHA	American heart association
Apo(a)	Apolipoprotein(a)
APR	Adjusted prevalence ratio
ARIC	Atherosclerosis Risk in Communities Study
ASCVD	Atherosclerotic cardiovascular disease
AST	Aspartate Aminotransferase
BMI	Body mass index
BP	Blood pressure
CGPS	Copenhagen General Population Study
CRP	C-reactive protein
DBP	Diastolic blood pressure
EAS	European Atherosclerosis Society
ELISA	Enzyme-linked immunosorbent assay
FBG	Fasting blood glucose
HbA1C	Glycosylated hemoglobin
HC	Hip circumference
HDL	High-density lipoprotein
HOMA-IR	Homeostasis model assessment for insulin resistance
HR	Hazard ratio
IQR	Interquartile range
KDEP	Kuwait Diabetes Epidemiological Program
KIV-2	Kringle IV type 2
LDL	Low-density lipoprotein
Lp(a)	Lipoprotein(a)
LT	Less than
MESA	Multi-Ethnic Study of Atherosclerosis
OR	Odds ratio
PR	Prevalence ratio
SBP	Systolic blood pressure
T2D	Type 2 diabetes mellitus
TC	Total cholesterol
TG	Triglycerides
WC	Waist circumference
